# Thermal Fabrication of Magnetic Fe_3_O_4_ (Nanoparticle)@Carbon Sheets from Waste Resources for the Adsorption of Dyes: Kinetic, Equilibrium, and UV–Visible Spectroscopy Investigations

**DOI:** 10.3390/nano13071266

**Published:** 2023-04-03

**Authors:** Mohamed A. Habila, Mohamed S. Moshab, Ahmed Mohamed El-Toni, Zeid A. ALOthman, Ahmed Y. Badjah Hadj Ahmed

**Affiliations:** 1 Department of Chemistry, College of Science, King Saud University, Riyadh 11451, Saudi Arabiazaothman@ksu.edu.sa (Z.A.A.);; 2 King Abdullah Institute for Nanotechnology, King Saud University, Riyadh 11451, Saudi Arabia; aamohammad@ksu.edu.sa

**Keywords:** nanoparticles, waste recycling, water treatment, adsorption, dye pollution, UV–visible spectroscopy

## Abstract

Thermal treatment is applied for the direct conversion of palm stalk waste to Fe_3_O_4_ (np)@carbon sheets (Fe_3_O_4_ (np)@CSs). The effect of conversion temperature was investigated. The TEM examination of the prepared magnetic Fe_3_O_4_ (np)@CSs showed the formation of Fe_3_O_4_ (np) in a matrix of carbon sheets as a coated layer with surface functional groups including carbonyl and hydroxyl groups. Removal of dyes such as methyl orange, methylene blue, and neutral red was achieved using fabricated Fe_3_O_4_ (np)@CSs which were prepared at 250 °C, 400 °C, and 700 °C in a weak acidic medium. By studying the contact time effect for the adsorption of methylene blue, neutral red, and methyl orange, using the fabricated Fe_3_O_4_ (np)@CSs which were prepared at 250 °C and 400 °C, equilibrium occurred between 120 min and 180 min. In addition, the first-order and second-order kinetic models were applied to the adsorption data. The results revealed that the adsorption data fit better with the second-order kinetic model. Furthermore, the Freundlich model was found to be more suitable for describing the process of the separation of the dyes onto Fe_3_O_4_ (np)@CSs which were prepared at 250 °C and 400 °C, suggesting heterogenous surfaces and multi-layer adsorption.

## 1. Introduction

Pollution is a global problem that is increasing day by day; therefore, it is necessary to assess this problem and develop solutions to prevent its associated negative impacts. As a result of water pollution, deaths and diseases occur all over the world [1,2,3]. The discharge of sewage and waste into water bodies poses significant threats to the pollution of lakes and rivers, in addition to garbage discharges, oil spills, and threats to the liquefaction capacities of lakes and rivers in major cities [4,5]. Water contamination with dyes constitutes a serious health problem and may cause toxicity or cancer [6,7,8].

Currently, the adopted techniques to control dye pollution include coagulation, co-sedimentation, ion exchange, membrane techniques, and adsorption processes. The purification methods must exhibit high efficiency, simplicity, low cost, and easy utilization of local resources. Adsorption is the most interesting technique, and many materials can be used as sorbents. Typical adsorbents are mainly activated carbon, metal oxides, and zeolites [9]. Low-cost materials represent a good alternative to the challenge of more widespread water treatment technologies. Recycling and reuse of waste are two energy-saving processes that have gained popularity due to their low cost and environmental friendliness. An example of these processes is the use of agricultural materials such as secondary waste resulting from various human activities [10,11,12,13].

Magnetic materials have great advantages as they can be easily rotated using an external magnetic field, and they can be controlled and directed towards the pollutant or separated from it [14]. One of the distinguishing properties is that the non-magnetic iron oxide can be reused after easy treatment. Easy reuse methods are possible by ultrasonic washing with hexane or ethanol within minutes [15]. According to recent studies, oil adsorbents can be used up to 1000 times [16]. Adsorption onto magnetic materials has biocompatibility properties suitable for organic materials, and the functional groups of organic materials can provide active sites for iron oxide particles, expanding their application processes in pollutant removal [17,18,19]. These functional groups play a major role in passivating iron oxide nanoparticles to prevent agglomeration before or after synthesis. Mostly, the polymers are chemically fixed or physically adsorbed on iron oxide nanoparticles to lower the surface tension to form Fe_3_O_4_ nanoparticles coated with a single or double organic layer [20,21]. There are many forms of iron oxides in nature. Magnetite (Fe_3_O_4_), hematite (α-Fe_2_O_3_), and maghemite (γ-Fe_2_O_3_) are the most common forms [22,23]. As a result of the inevitable problems that the bare iron oxide particles are exposed to, such as agglomeration and oxidation by air as a result of its high chemical activity, the very important magnetic property can be lost, preventing its application [24]. Magnetism prevents deterioration, including the iron oxide being covered with protective scales on its surface [25]. In previous years, it has been grafted and painted with organic and inorganic layers to protect it [26]. The reason for the efficient use of activated carbon as a coating layer around the magnetic core for efficient adsorption is due to the large number of surface pores that make the surface area subject to adsorption wider, relative to the actual effective volume, as well as providing the possibility of its recovery [27]. The magnetic carbon was developed by loading small particles of activated carbon on the surface of hydrated iron sulfate as a backing material. A group of researchers [27] prepared an effective adsorbent material by melting one of the polymeric compounds known as polyoxy2,6-dimethyl-1,4-phenylene with an n-caprolactam compound in the presence of some fine magnetic particles and used this substance in the adsorption of azo dyes prepared from triphenylmethane and some heterocyclic compounds from aqueous solutions. The study proved that there is a large variation in the adsorption of the selected dyes, and the dyes prepared from heterocyclic compounds demonstrated less adsorption. The conversion of biomass waste to carbon-based materials is a serious economic application as it is a renewable source, it is cost-effective, and there is the possibility of large-scale production [28,29,30,31,32]. Biomass, as a source of carbon during material processing, has been utilized to fabricate a tea waste/ Fe_3_O_4_ composite using a co-precipitation process for the removal of chromium (VI), with an adsorption capacity of 75.76 mg g^−1^. In addition, the coprecipitation process was applied to produce magnetic biochar from cotton which produced an adsorbent that promised separation efficiency for aniline removal [33]. Zhang et al. used Pt-nanoparticle-containing catalysts with a core–shell structure (Fe_3_O_4_-C-Pt) that were synthesized and applied for 5-hydroxymethylfurfural oxidation [34]. Anton et al. applied microwave-assisted fabrication of magnetic biochar for methylene blue removal [35]. Ferrone et al. applied a two-step method to fabricate Fe_3_O_4_–activated carbon from wastepaper, in which the Fe_3_O_4_ was fabricated first and then combined with the activated carbon of the wastepaper [36]. These methods consume more time and effort during the preparation procedures. Therefore, this study aimed to convert palm stalk waste to magnetic activated carbon using the direct thermal method to achieve the carbonization of waste simultaneously, with the formation of Fe_3_O_4_ magnetic nanoparticles (Fe_3_O_4_ (np)@CSs). In addition, this study aimed to investigate the potential of the fabricated Fe_3_O_4_ (np)@CSs for the adsorption of some dyes including methylene blue, methyl orange, and neutral red. Furthermore, we assessed the kinetic and isotherm models for the adsorption of the tested dyes onto the fabricated Fe_3_O_4_ (np)@CSs.

## 2. Materials and Methods

### 2.1. Fabrication of Fe_3_O_4_ (np)@CSs

The applied chemicals including iron (III) chloride hexahydrate, sulfuric acid, sodium hydroxide, hydrochloric acid, sodium dihydrogen phosphate, disodium hydrogen phosphate, methylene blue, methyl orange and neutral red were purchased from Sigma Aldrich, St. Louis, MO, USA. Palm stalk waste was collected from Riyadh city, divided into pieces between 5 cm and 10 cm, then washed with distilled water and dried in an oven at 105 °C. The palm stalk waste pieces were then ground to a fine powder. For the preparation of Fe_3_O_4_ (np)@CSs, a 10 g sample of the palm stalk fine powder was mixed with 6.75 g of iron (III) chloride hexahydrate in a sulfuric acid medium to form a paste-like mixture. The mixture was then heated in a muffle furnace at various temperatures of 250 °C, 400 °C, and 700 °C in the near absence of oxygen. Then, the formed Fe_3_O_4_ (np)@CSs were ground and washed with deionized water and ethanol several times. The obtained Fe_3_O_4_ (np)@CSs were examined by transmission electron microscopy (TEM), energy-dispersive spectroscopy (EDS), X-ray diffraction (XRD), and Fourier transform infrared spectroscopy (FTIR). Zeta potential analysis was applied to assess the surfaces charge variation of the prepared adsorbents including Fe_3_O_4_ (np)@CSs prepared at 250 °C, 400 °C, and 700 °C, with the pH variation from 2 to 12, using a Zeta nanosizer, Malvernpanalytical, Worcestershire, UK. A 0.05 g of Fe_3_O_4_ (np)@CSs prepared at 250 °C, 400 °C, and 700 °C was dispersed in 10 mL of NaOH (0.01 M), and the pH was adjusted to the desired pH values of 2, 4, 6, 8, 10, and 12 by adding drops of HCl or NaOH. The mixtures were exposed to ultrasonic waves for one minute, and then analyzed by zeta potential.

### 2.2. Adsorption Investigation for the Uptake of Dyes onto Fe_3_O_4_ (np)@CSs

The batch method was used to investigate the adsorption of methylene blue, methyl orange, and neutral red, as described in the previous literature [37,38]. Five hundred mg/L stock solutions from methylene blue, methyl orange, and neutral red were separately prepared. These stock solutions are diluted daily during adsorption studies. A 0.02 g of Fe_3_O_4_ (np)@CSs was mixed with 20 mL of methylene blue (100 mg/L), methyl orange (100 mg/L), and neutral red (100 mg/L) solution separately. The pH was controlled by adding 2 mL phosphate buffer solution and adjusted by adding drops of HCl or NaOH to the desired pH (2, 4, 5, 6, 8, 10, and 12). The mixtures were then shaken for 240 min at 150 rpm. Then, the Fe_3_O_4_ (np)@CSs adsorbents were separated from the aqueous medium by an external magnetic field, and the remaining dye solution was analyzed using UV–visible spectroscopy. The batch treatment processes were conducted in three replicates. In addition, the blank experiments were carried out at the same time during the dye adsorption processes. Then, the adsorption capacity for dye uptake was calculated by evaluating the dye concentration before and after the treatment process, using the following equation:

(1) $$q_{e}=\frac{\left(C_{0}-C_{e}\right)\times V}{M}$$

where C_0_ represents the dye initial concentration, C_e_ is the dye equilibrium concentration, V is the solution volume in liters, and M is the Fe_3_O_4_ (np)@CSs adsorbent mass in grams.

After investigation of the pH effect, pH 6 was selected for further study of methylene blue and methyl orange, while pH 2 was selected for further evaluation of neutral red. The previously described batch adsorption process was repeated to study the effect of contact time in the range of 10 min to 900 min at a dye concentration of 120 mg/L, an adsorbent dose of 0.02 g, a shaking rate of 150 rpm, and a temperature of 25 °C.

The effect of methylene blue, methyl orange, and neutral red dye concentration was investigated in the range 50 mg/L to 500 mg/L, at a contact time of 360 min, an adsorbent dose of 0.02 g, a shaking rate of 150 rpm, and a temperature of 25 °C. The adsorption data of methylene blue, methyl orange, and neutral red onto Fe_3_O_4_ (np)@CSs were subjected to kinetic and equilibrium modeling and compared with other adsorbents from the literature.

## 3. Results and Discussion

### 3.1. Morphology and Structural Characteristics of the Fabricated Fe_3_O_4_ (np)@CSs

**Figure 1:**
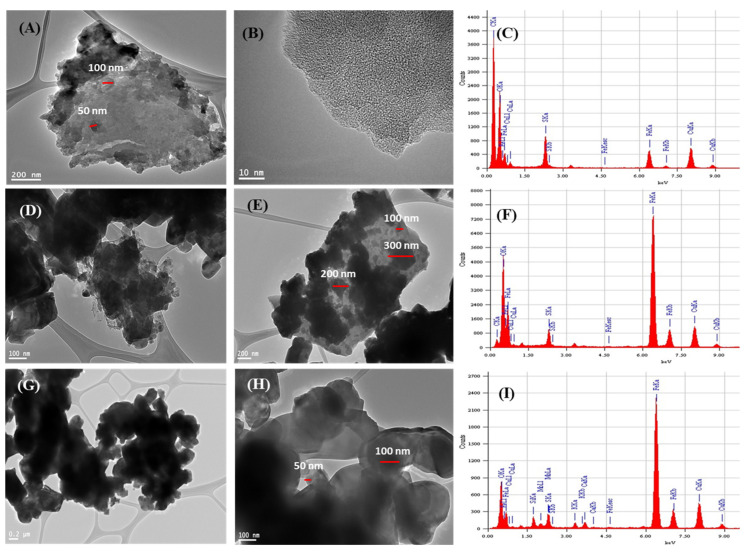
The TEM with various magnification and EDS examination for the magnetic Fe_3_O_4_ (np)@CSs prepared at 250 °C (**A**–**C**), at 400 °C (**D**–**F**), and at 700 °C, (**G**–**I**).

Activated carbon is a highly stable adsorbent material with a stable performance as an adsorbent in an acidic or basic medium. Herein, the combination of carbon materials with iron oxide was applied to produce Fe_3_O_4_ nanoparticles embedded into carbon sheets (Fe_3_O_4_ (np)@CSs). The produced nanocomposite exhibited magnetic properties which facilitate the separation of adsorbent using an external magnet after the adsorption of dyes. The nanocomposites of Fe_3_O_4_ as the core and carbon sheets as the shell improve the stability of Fe_3_O_4_ (np) during adsorption application and prevent leaching in an acidic medium. In addition, the whole particles of Fe_3_O_4_ (np)@CSs exhibit magnetic properties which enable easy filtration using the magnetic properties and avoid the difficulties associated with the adsorbent separation [39,40,41]. Herein, the high temperature was applied to enhance the formation of carbon sheets around the Fe_3_O_4_ nanoparticles. The temperature effect was investigated at 250 °C, 400 °C, and 700 °C. Figure 1 shows the TEM examination of the morphology and structure of a fabricated magnetic Fe_3_O_4_ (np)@CSs adsorbent. The preparation process at 250 °C produced an adsorbent material that poses high carbon content which is formed simultaneously with Fe_3_O_4_ (np) (Figure 1A,B). The formed Fe_3_O_4_ particles possess sizes between 50 nm to 100 nm. On the other hand, the heating at 400 °C during the preparation of magnetic Fe_3_O_4_ (np)@CSs led to the formation of well-coated Fe_3_O_4_ (np) with particle sizes between 100 nm to 300 nm which is embedded in carbon sheets as the shell, as presented in Figure 1D,E. The further increase in temperature at 700 °C, during the preparation of magnetic Fe_3_O_4_ (np)@CSs, led to the formation of Fe_3_O_4_ (np) with particles sizes between 50 nm and 100 nm, which are surrounded by a thin carbon shell due to the formation of ash and loosening parts of sample organic content, resulting in a poorly coated carbon shell (Figure 1G,H). By comparing the surface elemental ratio with the EDS, the main elements were found to be carbon, oxygen, and iron (Figure 1C,F,I). The carbon content was the highest in the case of samples prepared at 250 °C (Figure 1C), while the higher iron content was detected when the samples were heated to 400 °C (Figure 1F) and 700 °C (Figure 1I). This indicates that the applied heating conditions led to the successful formation of Fe_3_O_4_ (np)@CSs, most notably at higher temperatures of 400 °C and 700 °C. These results agreed with those of Hu et al., who reported the formation of pure Fe_3_O_4_ (np) at higher heating temperatures of 450 °C and 650 °C [42].

**Figure 2:**
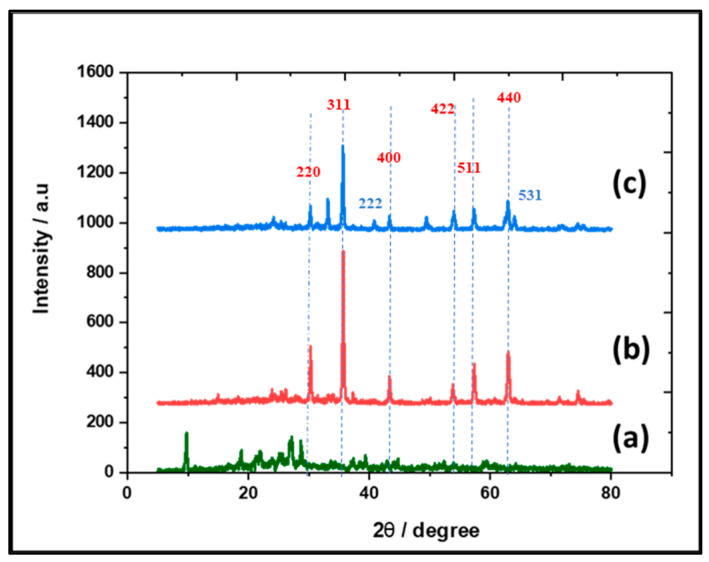
The XRD of the fabricated Fe_3_O_4_ (np)@CSs at (**a**) 250 °C, (**b**) 400 °C, and (**c**) 700 °C.

XRD was applied to identify the crystallinity in the formed structure of the fabricated Fe_3_O_4_ (np)@CSs at 250 °C, 400 °C, and 700 °C (Figure 2). It can be seen that the peaks relating to the Fe_3_O_4_ nanoparticles were detected in the fabricated samples at 400 °C (Figure 2b) which were similar to magnetite ((220), (311), (400), (511), and (440) planes). These results confirm the successful formation of a cubic crystal structure (Fe_3_O_4_ (JCPDS, 85-1436)) [42]. In contrast, under heat treatment at 700 °C (Figure 2c), some patterns in the plane of (222) and (531) were added, indicating the presence of a mixture of Fe_3_O_4_ and α Fe_2_O_3_ (np). However, the fabricated samples at 250 °C (Figure 2a) exhibited a lower crystalline structure, while the formed Fe_3_O_4_ (np)@CSs at 400 °C exhibited the clearest peaks.

**Figure 3:**
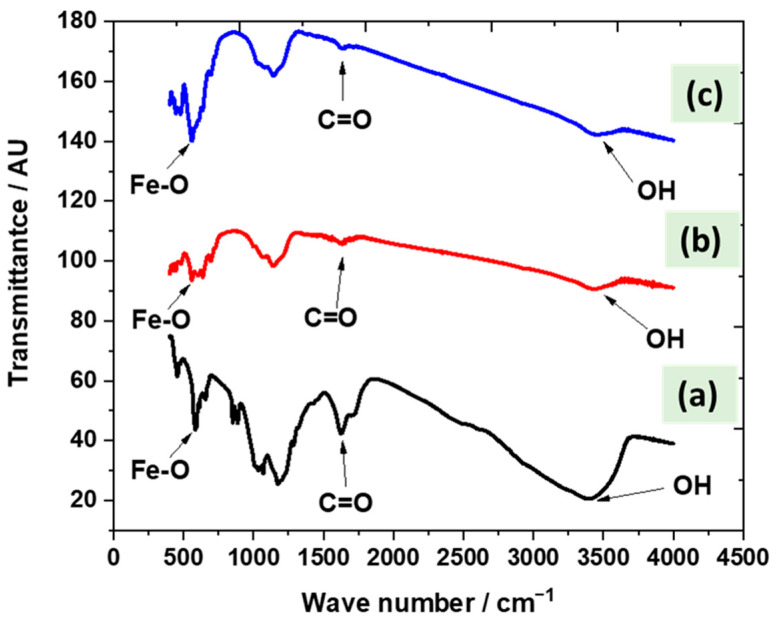
The FTIR of the fabricated Fe_3_O_4_ (np)@CSs at (**a**) 250 °C, (**b**) 400 °C, and (**c**) 700 °C.

Through FTIR analysis of the fabricated Fe_3_O_4_ (np)@CSs at 250 °C, 400 °C, and 700 °C, the peak related to the Fe–O bond in the structure of the Fe_3_O_4_ nanoparticles was detected in the three tested samples at approximately 570 cm^−1^. In addition, the carbonyl and hydroxyl groups appeared between 1600 cm^−1^ and 1700 cm^−1^ and 3300 cm^−1^ and 3450 cm^−1^, respectively, in the case of fabricated Fe_3_O_4_ (np)@CSs at 250 °C (Figure 3a). However, peaks related to the carbonyl and hydroxyl groups were reduced in the case of higher temperature fabrication (400 °C and 700 °C) (Figure 3b,c) due to carbonization and the formation of carbon sheets and iron oxide nanoparticles [43].

### 3.2. Evaluation of the Adsorption Capacity of the Fabricated Fe_3_O_4_ (np)@CSs Prepared at 250 °C, 400 °C, and 700 °C for the Removal of Dyes

**Figure 4:**
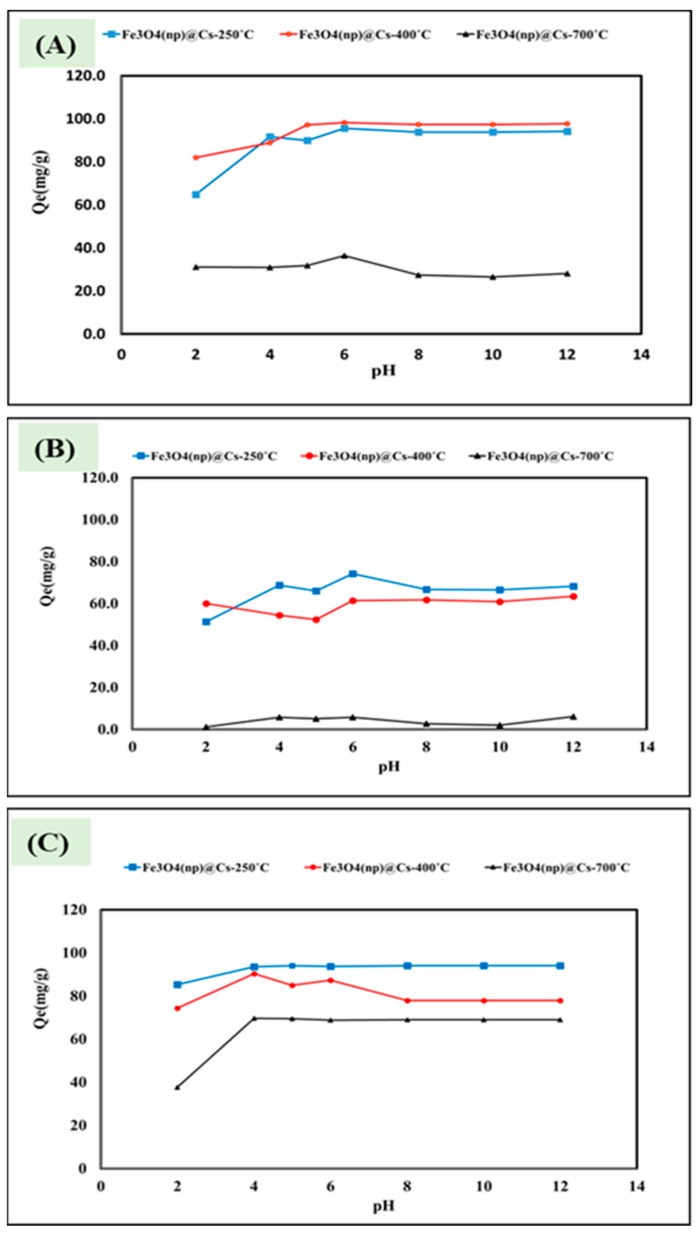
pH investigation for adsorption of (**A**) methylene blue, (**B**) neutral red, and (**C**) methyl orange onto magnetic Fe_3_O_4_ (np)@CSs prepared at 250 °C, 400 °C, and 700 °C. At a contact time of 240 min, a temperature of 25 °C, and 100 mg/L dye concentration.

**Figure 5:**
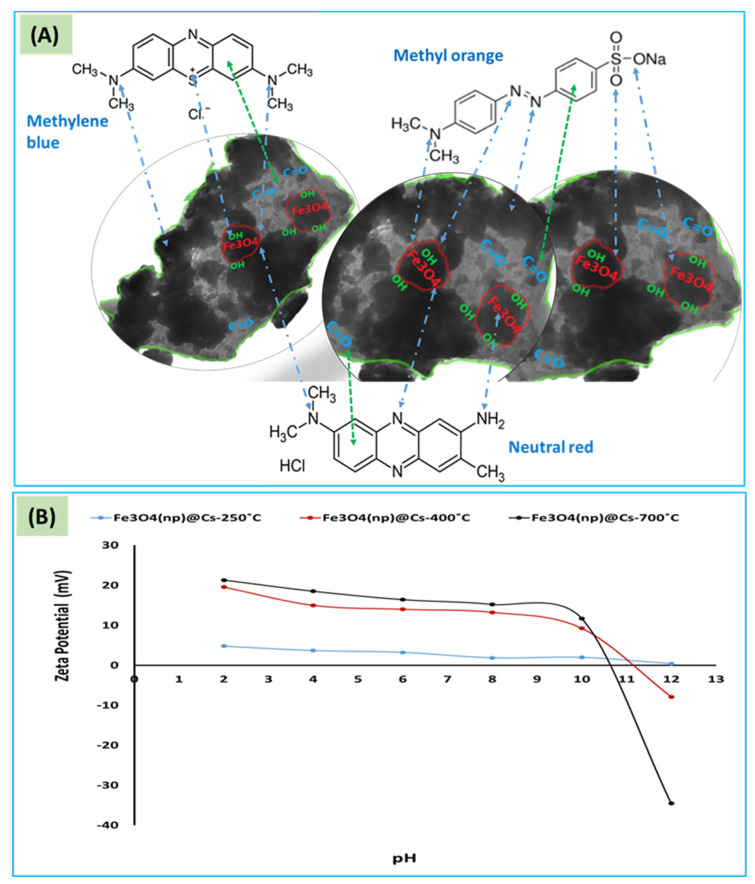
(**A**) Scheme for the adsorption of methylene blue, neutral red, and methyl orange onto magnetic Fe_3_O_4_ (np)@CSs, and (**B**) the zeta potential for magnetic Fe_3_O_4_ (np)@CSs prepared at 250 °C, 400 °C, and 700 °C.

The removal of dyes was applied to investigate the efficiency of the prepared magnetic carbon sheets as an adsorbent for water purification. The applied dyes included methylene blue, neutral red, and methyl orange. The pH of the adsorbent/adsorbate solution was studied from 2 to 12 and the adsorption capacity for the fabricated Fe_3_O_4_ (np)@CSs prepared at 250 °C, 400 °C, and 700 °C was calculated and is presented in Figure 4A–C. The fabricated Fe_3_O_4_ (np)@CSs prepared at 250 °C and 400 °C showed higher adsorption capacities compared with those prepared at 700 °C, which may be attributed to the loss of carbon content. In addition, the weak acidic medium was the most suitable for adsorption compared with the strong acidic or basic mediums, except for neutral red which possesses a higher adsorption capacity at pH 2 in the case of Fe_3_O_4_ (np)@CSs prepared at 400 °C (Figure 4B). The study of the effect of pH on adsorption was conducted by Anirudhan and Ramachandran (2015) using organoclay on the methylene blue (MB), reactive blue (RB), and crystal violet (CV) dye solutions. Their results showed that increasing the pH enhanced the adsorption capacity. A possible explanation for this is that increasing the pH reduces the positive charge on the surface and the clay surface becomes negatively charged, which favors the absorption of positive dyes due to electrostatic attraction [44]. When studying the effect of pH on the adsorption of divalent and trivalent heavy metal ions (nickel, lead, chromium, cadmium, copper, and manganese) on Na-montmorillonite, the researchers also expected the adsorption to decrease as the pH decreased because the silanol and aluminol groups were more protonated [45]. Herein, for the adsorption of methylene blue, neutral red, and methyl orange onto the Fe_3_O_4_ (np)@CSs prepared at 250 °C, 400 °C, and 700 °C, a possible explanation for the adsorption mechanism is provided in Figure 5A. The incorporation of iron oxide nanoparticles with the waste-derived carbon structure produces a heterogenous surface which is expected to enhance the electrostatic interaction with dyes to separate and move them from an aqueous solution to the surfaces of the Fe_3_O_4_ (np)@CSs, resulting in an effective adsorption process. The point of zero charge is important to illustrate the response of the adsorbent surfaces due to the pH change. The zeta potential has been applied to evaluate the effect of pH on the surface charge of the prepared Fe_3_O_4_ (np)@CSs prepared at 250 °C, 400 °C, and 700 °C. As presented in Figure 5B, by increasing the temperature during thermal treatment the surface electrostatic charge is increased with reporting of the lower surface charge for the materials prepared at 250 °C, which may be attributed to the higher carbon content. In addition, the overall surface charge remains positive until pH 10, reporting the point of zero charge between 10 and 12 for Fe_3_O_4_ (np)@CSs prepared at 400 °C and 700 °C, and for Fe_3_O_4_ (np)@CSs prepared at 250 °C, the surface charge became around 0.4 at pH 12. However, the adsorbent prepared at 700 °C showed higher surface electrostatic charge, and it exhibited the lowest adsorption capacity. This may be attributed to ash formation at a higher temperature which leads to lower carbon content, as confirmed by EDS examination (Figure 1I). The presence of carbon sheets decorated with carbonyl groups in the entire structure of the Fe_3_O_4_ (np)@CSs enabled the required matrix to carry and protect magnetic iron oxide nanoparticles, as well as the carbonyl active sites attracting dye molecules during adsorption [46,47,48]. The driving forces during the adsorption of methylene blue, neutral red, and methyl orange onto magnetic Fe_3_O_4_ (np)@CSs may involve Van der Waals forces, electrostatic interaction and dipole–dipole interaction between lone pairs of electrons in the dyes molecules and iron oxide structures which cause the positively charged Fe_3_O_4_ (np)@CSs surfaces, as well as between the carbonyl groups and S centers in methylene blue dye [49].

**Figure 6:**
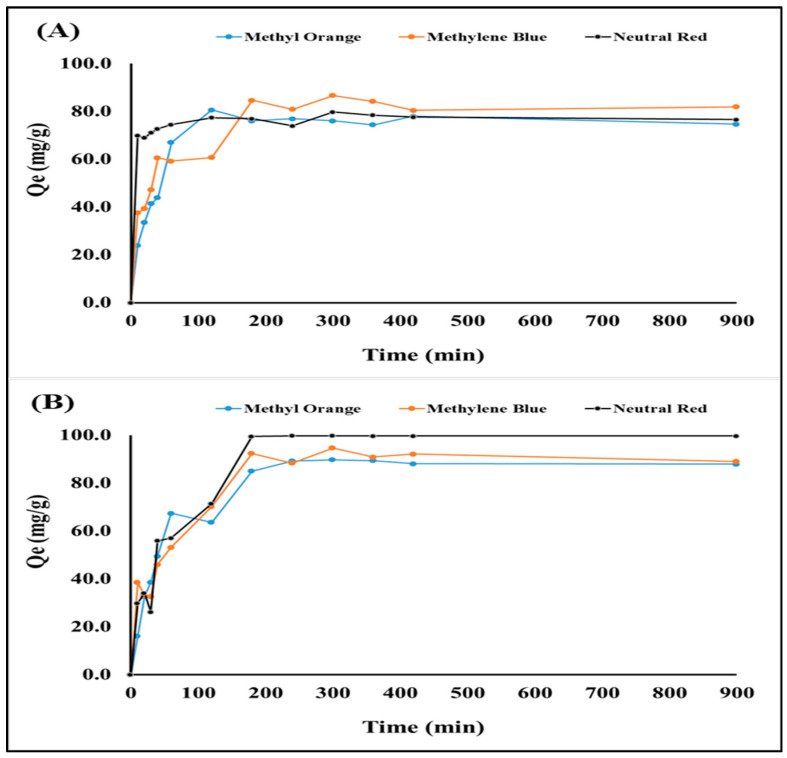
Studying the effect of contact time on adsorption of methylene blue (pH 4), neutral red (pH 2), and methyl orange (pH 4) onto magnetic Fe_3_O_4_ (np)@CSs prepared at (**A**) 250 °C and (**B**) 400 °C, at 25 °C, and 100 mg/L dye concentration.

By studying the contact time effect for the adsorption of methylene blue, neutral red, and methyl orange using the fabricated Fe_3_O_4_ (np)@CSs prepared at 250 °C and 400 °C, the equilibrium occurred at a time between 120 min and 180 min, as shown in Figure 6A,B. This can be attributed to the fabricated Fe_3_O_4_ (np)@CSs reaching a steady state due to the saturation of the Fe_3_O_4_ (np)CS adsorbent surfaces. The adsorption capacities of Fe_3_O_4_ (np)@ CSs-250 °C were 95 mg/g, 77 mg/g, and 93 mg/g for methylene blue, neutral red, and methyl orange, respectively, while for Fe_3_O_4_ (np)@ CSs-400 °C were 92 mg/g, 99 mg/g, and 90 mg/g for methylene blue, neutral red, and methyl orange, respectively. As is shown in Figure 6A,B, the prepared magnetic Fe_3_O_4_ (np)@CSs showed rapid adsorption of dyes; however, the adsorption capacity in the case of magnetic Fe_3_O_4_ (np)@CSs prepared at 400 °C was the best. The dye adsorption onto porous adsorbents may include three steps: adsorption on the outer material surfaces, dye diffusion into the internal pores, and adsorption onto the internal surfaces [50,51].

**Figure 7:**
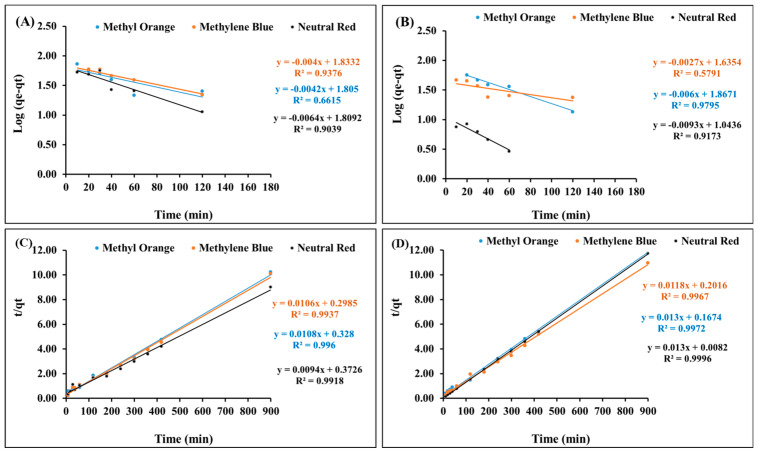
Kinetic investigations for the adsorption of methylene blue, neutral red, and methyl orange onto magnetic Fe_3_O_4_ (np)@CSs, first-order kinetics at (**A**) 250 °C and (**B**) 400 °C, and second-order kinetics at (**C**) 400 °C and (**D**) 250 °C.

In addition, the kinetic models of the pseudo-first-order and the pseudo-second-order kinetics were applied to the adsorption data, which fit better with the second-order kinetic model (Figure 7A–D). The pseudo-first-order (Equation (2)) [52,53] kinetic model was applied to investigate the rate of the adsorption of methylene blue, neutral red, and methyl orange using the fabricated Fe_3_O_4_ (np)@CSs prepared at 250 °C and 400 °C.

log(qe−qt) = log qe − K_1_t/2.303 (2)

Figure 7A,B presents the relationship between log(qe – qt) and t, and from the slope and intercept, the values of K_1_ and qe were calculated (Table 1).

The pseudo-second-order kinetic model in its integrated form of Equation (3) [54], was applied to investigate the rate of the adsorption of methylene blue, neutral red, and methyl orange using the fabricated Fe_3_O_4_ (np)@CSs prepared at 250 °C and 400 °C.

t/qt = (1/K_2_ qe^2^) + (1/qe) × T (3)

Figure 7C,D presents the relationship between t/qt and t, from which qe and K can be calculated (Table 1). The values of q experimental and q calculated showed a good correlation, indicating that the pseudo-second-order kinetic model is more suitable for describing the adsorption process. These results indicate a fast adsorption process [55].

The equilibrium status correlating to the concentrations of dyes with the adsorption capacities during the removal of methylene blue, neutral red, and methyl orange onto magnetic Fe_3_O_4_ (np)@CSs, according to the Langmuir and Freundlich models, was investigated.

The Langmuir Equation (4) was applied:

(4) $$\frac{C_{e}}{q_{e}}=(\frac{1}{Q_{\max}^{0}})C_{e}+\frac{1}{Q_{\max}^{0}K_{L}}$$

considering that the Q^0^_max_ (mg/g) is the maximum saturated monolayer adsorption capacity of the magnetic Fe_3_O_4_ (np)@CSs, Ce (mg/L) is the adsorbate concentration at equilibrium, qe (mg/g) is the amount of adsorbate uptake at equilibrium, and K_L_ (L/mg) is a constant associated with the affinity between the dyes and magnetic Fe_3_O_4_ (np)@CSs.

The correlation coefficient, R^2^, for the adsorption of methylene blue, neutral red, and methyl orange onto magnetic Fe_3_O_4_ (np)@CSs (Table 2) had lower values, revealing that the Langmuir model was not fitted with the adsorption data.

The Freundlich Equation (5) was applied:

Log qe = log K + 1/n log Ce(5)

where qe (mg/g) is the amount of adsorbate uptake at equilibrium, Ce (mg/L) is the adsorbate concentration at equilibrium, K_F_ (mg/g)/(mg/L) n is the Freundlich constant, and n (dimensionless) is the Freundlich intensity parameter.

**Figure 8:**
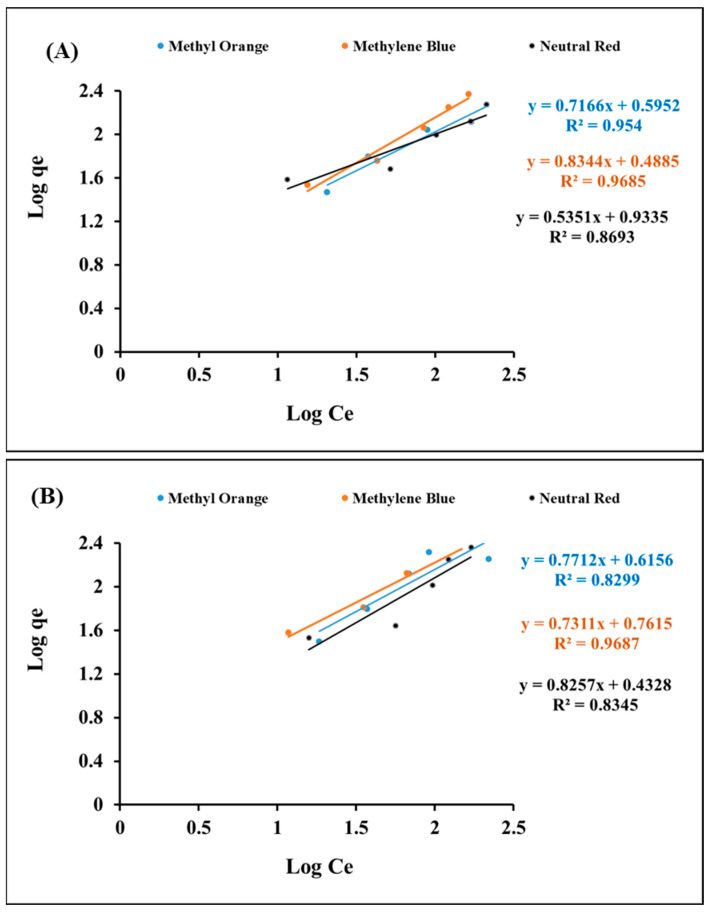
Freundlich isotherm for the adsorption isotherm of methylene blue, neutral red, and methyl orange. (**A**) Fe_3_O_4_ (np)@CSs-250 °C and (**B**) Fe_3_O_4_ (np)@CSs-400 °C.

The correlation coefficient, R^2^, for the adsorption of methylene blue, neutral red, and methyl orange onto magnetic Fe_3_O_4_ (np)@CSs (Figure 8A,B) (Table 2) revealed that the Freundlich model was found to be more suitable for describing the process of the separation of dyes onto Fe_3_O_4_ (np)@CSs prepared at 250 °C and 400 °C, especially in the case of methylene blue, suggesting the presence of heterogeneous surfaces and multi-layer adsorption.

The removal of dyes from wastewater has received significant efforts from scientists to reduce the negative environmental impacts of water pollution [56,57,58,59,60]. The adsorption capacity obtained in this work for the removal of methyl orange, methylene blue, and neutral red onto the fabricated Fe_3_O_4_ (np)@CSs was compared with others in the literature (Table 3) [61,62,63,64,65,66,67,68].

## 4. Conclusions

The thermal treatments at 250 °C, 400 °C, and 700 °C for palm stalk waste together with the iron (III) chloride hexahydrate in an acidic medium led to the formation of Fe_3_O_4_ (np)@carbon sheets (Fe_3_O_4_ (np)@CSs). The fabricated adsorbents of Fe_3_O_4_ (np)@CSs prepared at 250 °C, 400 °C, and 700 °C, exhibit positively charged surfaces under a pH range up to 10 as indicated from zeta potential measurements. However, the Fe_3_O_4_ (np)CS adsorbent prepared at 250 °C and 400 °C showed higher efficiency for the removal of methylene blue, neutral red, and methyl orange dyes. The thermal conditions at 400 °C were considered optimal for the formation of Fe_3_O_4_ in the entire structure of Fe_3_O_4_ (np)@CSs, as indicated by XRD. The fabricated Fe_3_O_4_ (np)@CSs at 250 °C and 400 °C showed high efficiency and fast performance for the removal of methylene blue, neutral red, and methyl orange. The adsorption capacities of Fe_3_O_4_ (np)@ CSs-250 °C were 95 mg/g, 77 mg/g, and 93 mg/g for methylene blue, neutral red, and methyl orange, respectively, while for Fe_3_O_4_ (np)@ CSs-400 °C, the adsorption capacities were 92 mg/g, 99 mg/g, and 90 mg/g for methylene blue, neutral red, and methyl orange, respectively. In addition, the reported adsorption process in this work is considered as a fast and effective method for the removal of dyes from wastewater. The practical and future applications of the prepared Fe_3_O_4_ (np)@CSs may include the adsorption of various pollutants such as pesticides, polychlorinated biphenyls, and/or heavy metals from wastewater. In addition, thermal treatment can be applied in future investigations to incorporate various metal oxide nanoparticles into waste-derived carbon for environmental and sustainable purposes.

## Figures and Tables

**Table 1:** The kinetic constant for adsorption of methylene blue, neutral red, and methyl orange using the fabricated Fe_3_O_4_ (np)@CSs prepared at 250 °C and 400 °C.

Adsorbent Material	Dye Name	qe, exp (mg/g)	Pseudo-First-Order Kinetics			Pseudo-Second-Order Kinetics		
			K_1_ (min^−1^)	qe, cal (mg/g)	R^2^	K_2_ (g/mg×min)	qe, cal (mg/g)	R^2^
Fe_3_O_4_ **(np)@ CSs 250 °C**	**Methyl orange**	80.7	9.6 × 10^−3^	63.8	0.66	1.01 × 10^−3^	76.9	0.99
	**Methylene blue**	84.7	9.2 × 10^−3^	68.1	0.93	6.9 × 10^−4^	84.7	0.99
	**Neutral red**	77.4	1.4 × 10^−3^	8.1	0.90	2.06 × 10^−2^	76.9	0.99
Fe_3_O_4_ **(np)@ CSs 400 °C**	**Methyl orange**	89.2	1.3 × 10^−2^	73.6	0.97	3.5 × 10^−4^	92.5	0.99
	**Methylene blue**	92.4	6.2 × 10^−3^	43.1	0.57	3.7 × 10^−4^	94.3	0.99
	**Neutral red**	99.5	2.1 × 10^−2^	11.5	0.91	2.7 × 10^−4^	106.3	0.99

**Table 2:** Langmuir and Freundlich isotherm constants for the adsorption of methylene blue, neutral red, and methyl orange using the fabricated Fe_3_O_4_ (np)@CSs prepared at 250 °C and 400 °C.

		Langmuir Constants			Freundlich Constants		
		K_L_ (L/mg)	Q max. (mg/g)	R^2^	K_F_ (mg/g)/(mg/L)^1/n^	n	R^2^
**Fe_3_O_4_ (np)@ CSs 250 °C**	Methyl orange	0.002703	5000	0.49	3.93	1.39	0.95
	Methylene blue	0.001658	10,000	0.25	3.07	1.19	0.96
	Neutral red	−0.0049	−10,000	0.74	8.85	1.33	0.86
**Fe_3_O_4_ (np)@ CSs 400 °C**	Methyl orange	0.000322	100,000	0.02	4.12	1.29	0.82
	Methylene blue	0.001873	10,000	0.24	5.77	1.36	0.96
	Neutral red	0.002688	1428	0.72	2.70	1.21	0.83

**Table 3:** Comparison of the adsorption capacities of the fabricated Fe_3_O_4_ (np)@CSs for this study and those reported in the literature for the removal of methyl orange, methylene blue, and neutral red.

Application Materials	Methyl Orange	Methylene Blue	Neutral Red	Reference
**(MMAC)**	98.5%	82%	-	[61]
**BiFeO_3_ nanoparticles**	4.06%	71.04%	98%	[62]
**Zeolitic imidazole frameworks (ZIFs)**	1340 mg/g	-	-	[63]
**ZIF-67 at Co-layered double hydroxide**	72.3%	79.9%	-	[64]
**Activated carbon prepared from rice husk residue**	63.8%	-	50.8–56.6%	[65]
**Fruit peels**	-	62.58 mg/g	-	[66]
**Micro/nanoscale magnesium silicate**	-	353 mg/g	-	[67]
**(β-CD/PAA/GO)**	-	247.99 mg/g	-	[68]
**Fe_3_O_4_ (np)@CSs-250 °C**	93 mg/g	95 mg/g	77 mg/g	This work
**Fe_3_O_4_ (np)@CSs-400 °C**	90 mg/g	92 mg/g	99 mg/g	This work

## Data Availability

The data presented in this study are available upon request from the corresponding author. The data are not publicly available due to privacy.

## References

[1] Duan W., He B., Nover D., Yang G., Chen W., Meng H., Zou S., Liu C. (2016). Water Quality Assessment and Pollution Source Identification of the Eastern Poyang Lake Basin Using Multivariate Statistical Methods. Sustainability.

[2] Saeed M.D., Mahmoud A.M. (2014). Determination of Some Physicochemical Parameters and Some Heavy Metals in Boreholes from Fagge LGA of Kano Metropolis Kano State-Nigeria. World J. Anal. Chem..

[3] Moe C.L., Rheingans R.D. (2006). Global challenges in water, sanitation and health. J. Water Health.

[4] McGauhey P.H. (1968). Engineering Management of Water Quality.

[5] Cariti F., Tuñas Corzon A., Fernandez-Cassi X., Ganesanandamoorthy P., Ort C., Julian T.R., Kohn T. (2022). Wastewater Reveals the Spatiotemporal Spread of SARS-CoV-2 in the Canton of Ticino (Switzerland) during the Onset of the COVID-19 Pandemic. ACS ES and T Water.

[6] Gonçalves M.M.M., Da Costa A.C.A., Leite S.G.F., Sant’Anna G.L. (2007). Heavy Metal Removal from Synthetic Wastewaters in an Anaerobic Bioreactor Using Stillage from Ethanol Distilleries as a Carbon Source. Chemosphere.

[7] Taştan B.E., Ertuğrul S., Dönmez G. (2010). Effective bioremoval of reactive dye and heavy metals by Aspergillus versicolor. Bioresour. Technol..

[8] Viggi C.C., Pagnanelli F., Cibati A., Uccelletti D., Palleschi C., Toro L. (2010). Biotreatment and bioassessment of heavy metal removal by sulphate reducing bacteria in fixed bed reactors. Water Res..

[9] Wang S., Wu H. (2006). Environmental-benign utilisation of fly ash as low-cost adsorbents. J. Hazard. Mater..

[10] Mohan D., Pittman C.U. (2007). Arsenic Removal from Water/Wastewater Using Adsorbents—A Critical Review. J. Hazard. Mater..

[11] Bansode R.R., Losso J.N., Marshall W.E., Rao R.M., Portier R.J. (2003). Adsorption of metal ions by pecan shell-based granular activated carbons. Bioresour. Technol..

[12] Kurniawan T.A., Chan G.Y.S., Lo W., Babel S. (2006). Comparisons of low-cost adsorbents for treating wastewaters laden with heavy metals. Sci. Total. Environ..

[13] Mehrasbi M.R., Farahmandkia Z., Taghibeigloo B., Taromi A. (2009). Adsorption of Lead and Cadmium from Aqueous Solution by Using Almond Shells. Water Air Soil Pollut..

[14] Zhang K., Mao L., Zhang L.L., Chan H.S.O., Zhao X.S., Wu J. (2011). Surfactant-intercalated, chemically reduced graphene oxide for high performance supercapacitor electrodes. J. Mater. Chem..

[15] Zhao G.X., Jiang L., He Y.D., Li J.X., Dong H.L., Wang X.K., Hu W.P. (2011). Sulfonated graphene for persistent aromatic pollutant management. Adv. Mater..

[16] Kadirvelu K., Faur-Brasquet C., Cloirec P. (2000). Le Removal of Cu (II), Pb (II), and Ni (II) by Adsorption onto Activated Carbon Cloths. Langmuir.

[17] Kikuchi Y., Qian Q., Machida M., Tatsumoto H. (2006). Effect of ZnO loading to activated carbon on Pb(II) adsorption from aqueous solution. Carbon.

[18] Peng X., Luan Z., Di Z., Zhang Z., Zhu C. (2005). Carbon nanotubes-iron oxides magnetic composites as adsorbent for removal of Pb(II) and Cu(II) from water. Carbon.

[19] Li Y.-H., Ding J., Luan Z., Di Z., Zhu Y., Xu C., Wu D., Wei B. (2003). Competitive adsorption of Pb2+, Cu2+ and Cd2+ ions from aqueous solutions by multiwalled carbon nanotubes. Carbon.

[20] Zhao G., Li J., Ren X., Chen C., Wang X. (2011). Few-Layered Graphene Oxide Nanosheets As Superior Sorbents for Heavy Metal Ion Pollution Management. Environ. Sci. Technol..

[21] Soenen S.J.H., Himmelreich U., Nuytten N., De Cuyper M. (2011). Cytotoxic effects of iron oxide nanoparticles and implications for safety in cell labelling. Biomaterials.

[22] Cornel R.M., Schwertmann U. (1996). The Iron Oxides. Structure, Properties, Reactions and Uses.

[23] Chan H.B.S., Ellis B.L., Sharma H.L., Frost W., Caps V., Shields R.A., Tsang S.C. (2004). Carbon-Encapsulated Radioactive99mTc Nanoparticles. Adv. Mater..

[24] Hummers W.S., Offeman R.E. (1958). Graphene Oxide. J. Am. Chem. Soc..

[25] Stankovich S., Dikin D.A., Dommett G.H.B., Kohlhaas K.M., Zimney E.J., Stach E.A., Piner R.D., Nguyen S.T., Ruoff R.S. (2006). Graphene-based composite materials. Nature.

[26] Myung S., Park J., Lee H., Kim K.S., Hong S. (2010). Ambipolar Memory Devices Based on Reduced Graphene Oxide and Nanoparticles. Adv. Mater..

[27] Šafařík I., Nymburská K., Šafaříková M. (1997). Adsorption of Water-soluble Organic Dyes on Magnetic Charcoal. Journal of Chemical Technology & Biotechnology: International Research in Process. Environ. Clean Technol..

[28] Qiang R., Feng S., Chen Y., Ma Q., Chen B. (2022). Recent progress in biomass-derived carbonaceous composites for enhanced microwave absorption. J. Colloid Interface Sci..

[29] Abuelnoor N., AlHajaj A., Khaleel M., Vega L.F., Abu-Zahra M.R.M. (2021). Activated carbons from biomass-based sources for CO2 capture applications. Chemosphere.

[30] Zhang H., Zhang Z., Luo J.-D., Qi X.-T., Yu J., Cai J.-X., Wei J.-C., Yang Z.-Y. (2019). A Chemical Blowing Strategy to Fabricate Biomass-Derived Carbon-Aerogels with Graphene-Like Nanosheet Structures for High-Performance Supercapacitors. ChemSusChem.

[31] Deng J., Li M., Wang Y. (2016). Biomass-derived carbon: Synthesis and applications in energy storage and conversion. Green Chem..

[32] Jain A., Balasubramanian R., Srinivasan M.P. (2016). Hydrothermal conversion of biomass waste to activated carbon with high porosity: A review. Chem. Eng. J..

[33] Gu Y., Xue Y., Zhang D. (2021). Adsorption of aniline by magnetic biochar with high magnetic separation efficiency. Environ. Pollut. Bioavailab..

[34] Zhang Y., Xue Z., Wang J., Zhao X., Deng Y., Zhao W., Mu T. (2016). Controlled deposition of Pt nanoparticles on Fe_3_O_4_@carbon microspheres for efficient oxidation of 5-hydroxymethylfurfural. RSC Adv..

[35] Zubrik A., Matik M., Lovás M., Štefušová K., Danková Z., Hredzák S., Václavíková M., Bendek F., Briančin J., Machala L. (2018). One-Step Microwave Synthesis of Magnetic Biochars with Sorption Properties. Carbon Lett..

[36] Ferrone V., Bruni P., Canale V., Sbrascini L., Nobili F., Carlucci G., Ferrari S. (2022). Simple Synthesis of Fe_3_O_4_@-Activated Carbon from Wastepaper for Dispersive Magnetic Solid-Phase Extraction of Non-Steroidal Anti-Inflammatory Drugs and Their UHPLC–PDA Determination in Human Plasma. Fibers.

[37] Habila M.A., Moshab M.S., Mohamed El-Toni A., Al-Awadi A.S., ALOthman Z.A. (2023). Facile Strategy for Fabricating an Organosilica-Modified Fe_3_O_4_ (OS/Fe_3_O_4_) Hetero-Nanocore and OS/Fe_3_O_4_@SiO_2_ Core–Shell Structure for Wastewater Treatment with Promising Recyclable Efficiency. ACS Omega.

[38] Alothman Z.A., Habila M.A., Al-Shalan N.H., Alfadul S.M., Ali R., Rashed I.G.A., Alfarhan B. (2016). Adsorptive removal of Cu(II) and Pb(II) onto mixed-waste activated carbon: Kinetic, thermodynamic, and competitive studies and application to real wastewater samples. Arab. J. Geosci..

[39] Corps Ricardo A.I., Sánchez-Cachero A., Jiménez-Moreno M., Guzmán Bernardo F.J., Rodríguez Martín-Doimeadios R.C., Ríos Á. (2018). Carbon nanotubes magnetic hybrid nanocomposites for a rapid and selective preconcentration and clean-up of mercury species in water samples. Talanta.

[40] Luo X., Lei X., Cai N., Xie X., Xue Y., Yu F. (2016). Removal of Heavy Metal Ions from Water by Magnetic Cellulose-Based Beads with Embedded Chemically Modified Magnetite Nanoparticles and Activated Carbon. ACS Sustain. Chem. Eng..

[41] Li D., He M., Chen B., Hu B. (2019). Metal organic frameworks-derived magnetic nanoporous carbon for preconcentration of organophosphorus pesticides from fruit samples followed by gas chromatography-flame photometric detection. J. Chromatogr. A.

[42] Hu P., Zhang S., Wang H., Pan D., Tian J., Tang Z., Volinsky A.A. (2011). Heat treatment effects on Fe_3_O_4_ nanoparticles structure and magnetic properties prepared by carbothermal reduction. J. Alloy. Compd..

[43] Habila M.A., Alothman Z.A., Mohamed El-Toni A., Labis J.P., Khan A., Al-Marghany A., Elafifi H.E. (2017). One-Step Carbon Coating and Polyacrylamide Functionalization of Fe_3_O_4_ Nanoparticles for Enhancing Magnetic Adsorptive-Remediation of Heavy Metals. Molecules.

[44] Carter C.L., Allen C., Henson D.E. (1989). Relation of Tumor Size, Lymph Node Status, and Survival in 24,740 Breast Cancer Cases. Cancer.

[45] Abollino O., Aceto M., Malandrino M., Sarzanini C., Mentasti E. (2003). Adsorption of heavy metals on Na-montmorillonite. Effect of pH and organic substances. Water Res..

[46] Hu X., Jia L., Cheng J., Sun Z. (2018). Magnetic ordered mesoporous carbon materials for adsorption of minocycline from aqueous solution: Preparation, characterization and adsorption mechanism. J. Hazard. Mater..

[47] Liu G., Li X., Campos L.C. (2017). Role of the functional groups in the adsorption of bisphenol A onto activated carbon: Thermal modification and mechanism. J. Water Supply Res. Technol..

[48] Xu J., Cao Z., Zhang Y., Yuan Z., Lou Z., Xu X., Wang X. (2018). A review of functionalized carbon nanotubes and graphene for heavy metal adsorption from water: Preparation, application, and mechanism. Chemosphere.

[49] Khalid K., Hanafiah M.A.K., Al-Amrani W.A., Malek N.A.N., Fatinathan S. (2022). Comparative Adsorption of Methylene Blue Dye on Hexane-Washed and Xanthated Spent Grated Coconut (*Cocos nucifera* L.): Isotherms, Thermodynamics and Mechanisms. J. Ecol. Eng..

[50] AlOthman Z.A., Habila M.A., Ali R., Abdel Ghafar A., Hassouna M.S. (2014). Valorization of two waste streams into activated carbon and studying its adsorption kinetics, equilibrium isotherms and thermodynamics for methylene blue removal. Arab. J. Chem..

[51] Habila M.A., Alothman Z.A., Ali R., Ghafar A.A., Hassouna M.S.E.-D. (2013). Removal of Tartrazine Dye onto Mixed-Waste Activated Carbon: Kinetic and Thermodynamic Studies. CLEAN–Soil Air Water.

[52] Wen D., Ho Y.-S., Tang X. (2006). Comparative sorption kinetic studies of ammonium onto zeolite. J. Hazard. Mater..

[53] Zhang Y.-Q., Dringen R., Petters C., Rastedt W., Köser J., Filser J., Stolte S. (2016). Toxicity of dimercaptosuccinate-coated and un-functionalized magnetic iron oxide nanoparticles towards aquatic organisms. Environ. Sci. Nano.

[54] Ho Y.S., McKay G., Wase D.A.J., Forster C.F. (2000). Study of the Sorption of Divalent Metal Ions on to Peat. Adsorpt. Sci. Technol..

[55] El-Toni A.M., Habila M.A., Ibrahim M.A., Labis J.P., Alothman Z.A. (2014). Simple and facile synthesis of amino functionalized hollow core–mesoporous shell silica spheres using anionic surfactant for Pb(II), Cd(II), and Zn(II) adsorption and recovery. Chem. Eng. J..

[56] Chen J., Xiong Y., Duan M., Li X., Li J., Fang S., Qin S., Zhang R. (2020). Insight into the Synergistic Effect of Adsorption–Photocatalysis for the Removal of Organic Dye Pollutants by Cr-Doped ZnO. Langmuir.

[57] Bhatti H.N., Safa Y., Yakout S.M., Shair O.H., Iqbal M., Nazir A. (2020). Efficient removal of dyes using carboxymethyl cellulose/alginate/polyvinyl alcohol/rice husk composite: Adsorption/desorption, kinetics and recycling studies. Int. J. Biol. Macromol..

[58] Zhu M.-X., Lee L., Wang H.-H., Wang Z. (2007). Removal of an anionic dye by adsorption/precipitation processes using alkaline white mud. J. Hazard. Mater..

[59] Yagub M.T., Sen T.K., Afroze S., Ang H.M. (2014). Dye and its removal from aqueous solution by adsorption: A review. Adv. Colloid Interface Sci..

[60] Liu Y., Zhao Y., Cheng W., Zhang T. (2020). Targeted reclaiming cationic dyes from dyeing wastewater with a dithiocarbamate-functionalized material through selective adsorption and efficient desorption. J. Colloid Interface Sci..

[61] Azam K., Raza R., Shezad N., Shabir M., Yang W., Ahmad N., Shafiq I., Akhter P., Razzaq A., Hussain M. (2020). Development of recoverable magnetic mesoporous carbon adsorbent for removal of methyl blue and methyl orange from wastewater. J. Environ. Chem. Eng..

[62] Mohd Kaus N.H., Imam S.S., Aziz A.W., Lee H.L., Adnan R., Ibrahim M.L. (2021). Controlled growth of BiFeO_3_ nanoparticles in the presence of alginate template for adsorptive removal of different dyes. Colloids Surfaces A Physicochem. Eng. Asp..

[63] Zhang Z.-H., Zhang J.-L., Liu J.-M., Xiong Z.-H., Chen X. (2016). Selective and Competitive Adsorption of Azo Dyes on the Metal–Organic Framework ZIF-67. Water Air Soil Pollut..

[64] Nazir M.A., Khan N.A., Cheng C., Shah S.S.A., Najam T., Arshad M., Sharif A., Akhtar S., Rehman A.U. (2020). Surface induced growth of ZIF-67 at Co-layered double hydroxide: Removal of methylene blue and methyl orange from water. Appl. Clay Sci..

[65] Li Y., Zhang X., Yang R., Li G., Hu C. (2016). Removal of dyes from aqueous solutions using activated carbon prepared from rice husk residue. Water Sci. Technol..

[66] Mallampati R., Xuanjun L., Adin A., Valiyaveettil S. (2015). Fruit Peels as Efficient Renewable Adsorbents for Removal of Dissolved Heavy Metals and Dyes from Water. ACS Sustain. Chem. Eng..

[67] Zhang J., Dang L., Zhang M., Zhao S., Lu Q. (2017). Micro/nanoscale magnesium silicate with hierarchical structure for removing dyes in water. Mater. Lett..

[68] Liu J., Liu G., Liu W. (2014). Preparation of water-soluble β-cyclodextrin/poly(acrylic acid)/graphene oxide nanocomposites as new adsorbents to remove cationic dyes from aqueous solutions. Chem. Eng. J..

